# Cross-Sectional Study on the Association between Dietary Non-Enzymatic Antioxidant Capacity and Serum Liver Enzymes: The Furukawa Nutrition and Health Study

**DOI:** 10.3390/nu12072051

**Published:** 2020-07-10

**Authors:** Hinako Nanri, Ikuko Kashino, Takeshi Kochi, Masafumi Eguchi, Shamima Akter, Akiko Nanri, Isamu Kabe, Tetsuya Mizoue

**Affiliations:** 1 Department of Epidemiology and Prevention, Center for Clinical Sciences, National Center for Global Health and Medicine, Shinjuku-ku, Tokyo 162-8655, Japan; ikukomax2007@yahoo.co.jp (I.K.); sakter@hosp.ncgm.go.jp (S.A.); nanri@fwu.ac.jp (A.N.); mizoue@hosp.ncgm.go.jp (T.M.); 2 Department of Health Administration, Furukawa Electric Corporation, Tokyo 100-8322, Japan; takeshi.kochi@furukawaelectric.com (T.K.); masafumi.eguchi@furukawaelectric.com (M.E.); isamu.kabe@kubota.com (I.K.); 3 Department of Food and Health Sciences, International College of Arts and Sciences, Fukuoka Women’s University, Fukuoka 813-8529, Japan; 4 Kubota Corporation, Tokyo 104-8307, Japan

**Keywords:** non-enzymatic antioxidant capacity, liver enzymes, Japanese

## Abstract

We examined the association of dietary non-enzymatic antioxidant capacity (NEAC) in overall diet, and separately from foods and beverages, with serum liver enzymes in a Japanese working population. This cross-sectional study was conducted among 1791 employees aged 18–69 years, who underwent a comprehensive health checkup in 2012–2013. A brief validated self-administered diet-history questionnaire was used for dietary assessment, and dietary NEAC intake was determined from databases of NEAC values, obtained using ferric reducing-antioxidant power (FRAP) and oxygen radical absorbance capacity (ORAC) assays. The dietary NEAC intake was calculated by multiplying the estimated NEAC values by the amounts consumed and summing the resulting values. A multiple-regression analysis was performed to explore the association between dietary NEAC intake and the serum levels of liver enzymes (aspartate aminotransferase (AST), alanine aminotransferase (ALT), and gamma-glutamyltransferase (GGT)), after adjustment for confounding factors. No significant associations were found between overall dietary NEAC intake and AST (FRAP, *p* for trend = 0.97; ORAC, *p* = 0.72), ALT (FRAP, *p* = 0.73; ORAC, *p* = 0.92), and GGT (FRAP, *p* = 0.96; ORAC, *p* = 0.19) levels. Food-derived, but not beverage-derived, NEAC intake was inversely associated with serum GGT levels (FRAP, *p* for trend = 0.001; ORAC, *p* = 0.02), particularly among older participants and those with high serum ferritin concentrations. The results imply that overall dietary NEAC intake is not associated with liver dysfunction, and that the NEAC values from foods may be inversely associated with serum GGT levels.

## 1. Introduction

Oxidative stress, which is defined as the imbalance between the production of reactive oxygen species (ROS) in cells and tissues and antioxidant species, is a crucial factor for chronic disease [1,2]. Evidence has been accumulated that demonstrates that excess production of ROS causes cell death through necrotic and/or apoptotic mechanisms, leading to cellular and tissue injury [3]. Thus, the reduction of oxidative stress by dietary antioxidants (e.g., carotene and vitamin C) may protect against cell damage [4,5], by suppressing the overproduction of ROS or inhibiting the reactions that involve ROS [6]. Given that people typically consume complex combinations of antioxidants found in a variety of foods, it is important to investigate the effects of antioxidants widely consumed from the diet. Recently, the non-enzymatic antioxidant capacity (NEAC), which considers the synergistic interactions of redox molecules in the complex matrices of food and biological sample, has been proposed as an effective new tool [7]. Using this approach, epidemiology studies show that high dietary NEAC is inversely associated with oxidative stress-related diseases [8,9].

Previous results from observational studies have reported that a higher dietary intake [10] or higher blood concentrations [11] of antioxidant nutrients were associated with decreased serum liver enzyme levels, particularly gamma-glutamyltransferase (GGT) which is a major biomarker of excessive alcohol intake [12] and oxidative stress [13]. To date, several [10,14] but not all [11] studies have reported that intakes of fruits and vegetables, which are rich in antioxidants, were inversely associated with liver enzymes levels. Although green tea contains high concentrations of catechin-type polyphenol antioxidants, studies of the effects of green tea consumption on liver dysfunction have yielded inconsistent results [15,16,17,18]. However, epidemiological evidence on the association between dietary NEAC intake and serum liver enzyme levels is sparse [19]. Moreover, given that the effect of plant-based foods on plasma NEAC is more prominent than that of beverages in healthy subjects [20], the bioavailability of antioxidants on liver dysfunction may differ depending on the dietary source.

The objective of this study was to examine whether dietary NEAC intake is associated with serum liver enzyme levels, and whether the association differs according to the dietary source of the NEAC intake (food or beverages) in a Japanese working population. We also investigated whether the association was modified by risk factors for liver injury, including age, body mass index (BMI), smoking, alcohol drinking, and serum ferritin levels. We hypothesized that dietary NEAC would be inversely associated with serum liver enzymes.

## 2. Subjects and Methods

### 2.1. Study Procedure and Participants

In April 2012 and May 2013, the present cross-sectional study was derived from the Furukawa Nutrition and Health Study, as described elsewhere [21,22]. In brief, a health survey was conducted during the periodic health examinations of workers from two locations of a manufacturing corporation and its affiliated companies in Chiba Prefecture and Kanagawa Prefecture. Prior to the health checkup, all employees of the companies were invited to complete two questionnaires—one for diet and the other for health-related lifestyle. Of the 2828 checkup attendees, 2162 agreed to participate in the study (response rate, 76%). We also obtained health checkup data, including the results of anthropometric and biochemical parameters and information on history of disease. The study protocol has been approved by the Ethics Committee of the National Center for Global Health and Medicine (NCGM-G-001140-26), and written informed consent was obtained from all the participants.

Of the 2162 participants, we excluded 11 who did not return the questionnaires; 148 who lacked data on serum liver enzyme levels; 110 with a history of hepatitis B or hepatitis C, cancer, cardiovascular disease, nephritis, or diabetes; and 91 with missing information on the covariates included in the analysis. We also excluded 11 participants who reported an extreme total energy intake (outside the mean ± 3 standard deviations [SD]). Thus, 1791 participants (1620 males and 171 females) were included in the analysis.

### 2.2. Blood Sampling and Laboratory Assays

Laboratory assays were performed with two different laboratory test companies. Serum aspartate aminotransferase (AST), alanine aminotransferase (ALT), and GGT levels were measured by the Japan Society of Clinical Chemistry recommended method, using a JCA-BM8060 (JEOL, Ltd., Tokyo, Japan) for the 2012 survey, and an AU5400 (Beckman Coulter, Inc., Brea, CA, USA) for the 2013 survey. Pureauto S AST, Pureauto S ALT, and Pureauto S GGT (Sekisui Medical Co., Tokyo, Japan) for the 2012 survey and Cica Luquid AST, Cica Liquid ALT and Cica Luquid GGT (Kanto Chemical Co., Tokyo Japan) for the 2013 survey were used as reagents. Serum ferritin levels were measured by a chemiluminescence immunoassay kit (Chemilumi ACS-Ferritin II; Siemens Healthcare Diagnostics, Tokyo, Japan) on an ADVIA Centaur (Bayer, Tarrytown, NY, USA).

### 2.3. Dietary Intake

Dietary habit during the preceding one-month period was assessed using a validated brief validated self-administered diet-history questionnaire (BDHQ) [23]. The consumption frequency of 58 food and beverage items, energy, and selected nutrients were estimated using an ad hoc computer algorithm for the BDHQ [24], with reference to the Standard Tables of Food Composition in Japan [25]. To estimate dietary NEAC intake levels, we assigned an NEAC value to each food item in the BDHQ; the details regarding the estimation have been described elsewhere [9,26], using the following two assays: (i) ferric reducing antioxidant power (FRAP), which measures the ability of an antioxidant to reduce Fe^3+^ (ferric ion) to Fe^2+^ (ferrous iron); (ii) oxygen radical absorbance capacity (ORAC), which measures the chain-breaking capacity of an antioxidant’s scavenging activity against peroxyl radicals, and the area under the fluorescence decay curve over time [27]. Dietary NEAC of processed foods were calculated from the NEAC values of individual ingredients and their proportions within the food using the food component, based on the Standard Tables of Food Composition in Japan [25]. As with previous studies [26,28,29,30], we did not take into account the NEAC value of coffee, because it is unclear whether high-molecular-weight antioxidants are absorbed in vivo [31]. Maillard products, which are produced during coffee roasting, are the major contributors to the in vitro antioxidant capacity of coffee [32]. The dietary NEAC intake was calculated by multiplying the NEAC values of individual foods and beverages by the amounts consumed and summing the resulting values for each participant.

### 2.4. Other Variables

Each subject’s height and weight were assessed to the nearest 0.1 cm and 0.1 kg, respectively, and the BMI was calculated by dividing weight by squared height (kg/m^2^). Occupational (including domestic housework and commuting) and leisure-time physical activities, smoking status, alcohol drinking, dyslipidemia, use of medication, and use of vitamin or mineral supplements were identified from the self-administered questionnaire. Occupational and leisure-time physical activities were evaluated as the sum of metabolic equivalents (METs), multiplied by the duration (in hours) across physical activities with different levels. Iron stores were evaluated by measuring serum ferritin levels.

### 2.5. Statistical Analysis

Dietary NEAC intake was adjusted for energy using the density method. We calculated the percentage contribution of each item (food and beverage) to the overall dietary NEAC intake. To identify the major contributors to NEAC intake, we categorized the contribution to the overall NEAC intake of each food item. We also categorized the energy-adjusted dietary NEAC intake into quartiles. Participants’ characteristics are expressed as means (SD) or medians (25th percentile, 75th percentile) for continuous variables and percentages for categorical variables. Trend associations between categories of dietary NEAC and confounding variables were subjected to linear regression analysis or Wilcoxon’s rank test for continuous variables and the Mantel–Haenszel chi-squared test for categorical variables; we assigned ordinal numbers 0–3 to the dietary NEAC categories. We used a multiple regression analysis to examine the associations of overall dietary NEAC intake, NEAC from foods, and NEAC from beverages with serum liver enzyme levels. Because all the liver enzymes tested were positively skewed, they were log-transformed to better approximate a normal distribution. Multiple linear regression was used to estimate adjusted geometric means and their 95% confidence intervals (CIs) for the quartiles of dietary NEAC. We adjusted for age (year, continuous), sex, workplace (site A or site B), BMI (kg/m^2^, continuous), occupational physical activity (<3, 3 to <7, 7 to <20, or ≥20 METs-hour/day), leisure-time physical activity (0, >0 to <3, 3 to <10, or ≥10 METs-hour/week), smoking (never-smoker, former smoker, current smoker of <20 cigarettes/day, or current smoker of ≥20 cigarettes/d), alcohol drinking (drinker consuming <1 d/wk, drinker consuming <23 g of ethanol/day, drinker consuming ≥23–<46 g of ethanol/d, or drinker consuming ≥46 g of ethanol/d), dyslipidemia (yes or no), non-steroidal anti-inflammatory drugs (yes or no), log-transformed serum ferritin level (μg/L, continuous), use of supplements (vitamin E, vitamin C, or multivitamins; yes or no), coffee consumption (<1, 1, or ≥2 cups/day), and total energy intake (kcal/day, continuous). Trend associations were assessed using multiple linear regression analysis, with the ordinal numbers 0 to 3 assigned to each quartile category of dietary NEAC. We also performed analyses stratified according to median age (<42 or ≥42 years), BMI (<25 or ≥25 kg/m^2^), smoking status (non-smoker or smoker), alcohol drinking (<23 or ≥23 g of ethanol/d), and median serum ferritin level (<155 or ≥155 μg/L for males and <23 or ≥23 μg/L for females). An interaction term was generated by multiplying the dietary NEAC intake (continuous) by the above dichotomized variables and adding it to the multivariate model. Two-sided *p* values of less than 0.05 were regarded as indicative of statistical significance. The statistical analysis was performed using Statistical Analysis System (SAS) software, version 9.4 (SAS Institute, Inc., Cary, NC, USA).

## 3. Results

The median dietary NEAC intake was 3.08 μmol Fe^2+^/day by FRAP and 4.72 μmol TE/day by ORAC. The median AST, ALT, and GGT values were 21.0, 22.0, and 29.0 U/L, respectively. As shown in Table 1, the percentage contributions of the food items to dietary NEAC intake are listed. The major food items that contributed to dietary NEAC intake were green tea (FRAP, 38.1%; ORAC, 35.2%), vegetables (FRAP, 17.4%; ORAC, 21.8%), and black and oolong tea (including other Chinese teas) (FRAP, 14.3%; ORAC, 13.3%). Based on the overall NEAC quartiles, beverages, particularly green tea, made the greatest contribution to the total NEAC intake in each quartile, with the exception of the first quartile (Supplemental Figure S1).

The characteristics of study participants, according to quartiles categories of dietary NEAC, are shown in Table 2. Participants with a higher dietary NEAC intake tended to be older, have a history of dyslipidemia, engage in physical activity in their leisure time, and have a higher intake of antioxidant vitamins. However, they were less likely to be male, current smoke, and alcohol drinkers (ORAC only) and to have lower work-related physical activity, serum ferritin concentrations, and coffee and energy intake values.

Table 3 shows the adjusted geometric mean serum liver enzyme levels and their 95% CIs according to the NEAC quartile. Overall, the total NEAC intake was not significantly associated with serum AST (FRAP, *p* for trend = 0.97; ORAC, *p* = 0.72), ALT (FRAP, *p* = 0.73; ORAC, *p* = 0.92), or GGT (FRAP, *p* = 0.96; ORAC, *p* = 0.19) levels after adjustment for all covariates. The NEAC from foods was significantly inversely associated with serum GGT levels (FRAP, *p* for trend = 0.001; ORAC, *p* = 0.02), but not with serum AST or ALT levels. In contrast, the NEAC from beverages was not associated with any serum liver enzyme.

In analyses by risk factors for liver injury (i.e., age, BMI, smoking, alcohol drinking, and serum ferritin level), there was no significant interaction of the overall dietary NEAC intake estimated by FRAP (Table 4) and ORAC (Supplemental Table S1) with risk factors for liver injury. Analyses of NEAC (FRAP) by different sources detected statistically significant interactions between age or serum ferritin status and NEAC from foods for GGT levels (*p* for interaction <0.05; Supplemental Table S2). We found that serum GGT levels were lower with increasing NEAC from foods in the older (*p* for trend = 0.001) and high-ferritin-level (*p* = 0.004) groups, but not in the younger (*p* = 0.32) or low-ferritin-level (*p* = 0.16) groups. For ORAC, the results revealed a marginally significant interactive effect on the GGT levels of serum ferritin levels and the NEAC from foods *(p* for interaction = 0.06, Supplemental Table S3).

## 4. Discussion

In this cross-sectional study of Japanese workers, we observed no association between overall dietary NEAC intake and serum AST, ALT and GGT. In analyses by dietary NEAC source, neither higher dietary NEAC from foods nor beverages were associated with serum AST and ALT. Additionally, dietary NEAC from foods, but not from beverages, was inversely associated with serum GGT levels, only in older participants and those with high ferritin levels. To our knowledge, this is the first observational study of the associations between dietary NEAC intake and serum levels of liver enzymes.

High dietary NEAC has been suggested to reduce oxidative stress, an important cause of liver dysfunction. Contrary to our expectation, we did not observe an inverse association between dietary overall NEAC and liver enzymes in the present study. We repeated the analysis in men only (*n* = 1620, proportion of men: 90.5%); however, the results did not materially change. To our knowledge, there was no observational study of this relationship. A small intervention study among 33 healthy adults reported a favorable effect of NEAC diet (fruits, vegetables, coffee, and tea were the major contributors) on serum ALT and GGT, but not AST [19] Similarly, a cross-sectional study of 265 Tehrani healthy adults have reported that vegetable intake was inversely associated with serum ALT, but not AST [14]. In contrast, green tea consumption was not associated with serum AST and ALT in healthy Japanese adults [16].

Our result on the inverse association of NEAC from foods with serum GGT is consistent with prior reports on the association of fruits and vegetables (rich in antioxidants) with serum GGT levels [10,33]. A prospective study in the United States showed that higher intake of fruits at baseline was associated with lower serum GGT levels after 10 years [10], and a Japanese cross-sectional study reported lower serum GGT levels among subjects with higher intake of fruits and vegetables [33]. Our result is also supported by a cross-sectional study in Japan, which showed that lower GGT levels were associated with a healthy dietary pattern characterized by a high intake of vegetables and fruits [34]. Notably, an association with the NEAC from foods was confined to older participants and those with high serum ferritin levels, both of which are related to increased oxidative stress [35,36]. This can be caused by inadequate endogenous redox defenses in high-risk individuals, who thus require exogenous antioxidants to maintain the redox balance. Further research is required to determine whether the effect of dietary NEAC intake on serum GGT differs according to oxidative status.

Our result of no association between dietary NEAC from beverages and serum GGT is in line with the present observational studies—two Japanese cross-sectional studies on the relationship of green tea consumption with serum GGT levels [15,16]. Likewise, a cross-sectional study involving healthy Japanese males reported that polyphenol intake from green tea was not associated with serum GGT levels [37]. The null finding of the present study may also be due to the low bioavailability of antioxidants obtained from beverages compared to foods. Although green tea is a food rich in polyphenols [38], in vivo, the fate of dietary polyphenols in the body shows a low degree of absorption [39]; the blood concentrations of polyphenols (e.g., catechins), which are present mainly in beverages, were lower than those of other antioxidants (e.g., ascorbic acid, which is abundant in fruits and vegetables) [39,40]. In addition, a meta-analysis of dietary intervention studies showed that the effect of antioxidant-rich beverages (such as green tea and black tea) on plasma NEAC was of lower magnitude than was that of plant-based foods in healthy participants [20]. Thus, our results do not support the hypothesis that the NEAC from beverages reduces the serum levels of GGT.

The major strengths of this study include the high participation rate, the use of a validated dietary questionnaire (BDHQ), the exclusion of individuals with a history of chronic disease, and adjustment for the potential confounding factors of liver injury. However, this study also has several limitations. First, an association derived from a cross-sectional study cannot be used to make causal inferences. However, we endeavored to minimize the effect of reverse causality, by excluding participants with chronic diseases that might have affected their dietary habits. Second, dietary NEAC values were estimated using not only Japanese databases, but also those from other countries, due to the non-availability of NEAC values for some Japanese food and beverage items. Given that the antioxidant content of foods varies geographically [41], different results might have been obtained if we had used NEAC values from only Japanese databases. Third, we could not estimate NEAC value for foods and beverages not included in the BDHQ. Thus, dietary NEAC values might be underestimated compared with true dietary NEAC intake. In addition, dietary intake was assessed at only one point in time, and thus may not reflect long-term intake. Fourth, given that blood levels of NEAC are determined by various factors including endogenous antioxidants [20], dietary NEAC estimation may not be equal physiologically to active NEAC. Further studies are required to confirm the association between dietary NEAC and the endogenous redox network and their impact on serum liver enzymes. Fifth, the possibility of bias due to residual confounding and unmeasured factors cannot be ruled out. For example, information regarding non-alcoholic fatty liver disease and alcoholic liver disease, which are known risk factors for elevated levels of ALT and AST [12,42], were not obtained in our study. Fifth, the study participants were almost exclusively men (*n* = 1625, 90.5%). Additionally, the study was conducted in selected workplaces; hence, the results cannot be applied to the general population.

## 5. Conclusions

We observed no association between overall dietary NEAC intake and the serum levels of liver enzymes. Dietary NEAC from foods, but not from beverages, was inversely associated with serum GGT levels. Further prospective studies are needed to confirm these associations.

## Figures and Tables

**Table 1:** The percentage contributions of the food and beverage items to dietary NEAC intake *^a.^*

Food Group and Item *^b^*	% Total NEAC by FRAP *^c^*	% Total NEAC by ORAC
Food group		
Vegetables	17.4	21.8
Fruits	5.4	8.4
Cereals	3.8	3.7
Pulses	2.4	7.5
Oil	2.1	-
Confectionary	1.5	0.1
Potatoes	1.1	3.7
Beverage Group		
Green Tea	38.1	35.2
Black and Oolong Tea	14.3	13.3
Alcoholic Beverages	9.1	1.2
Fruit Juice and Vegetable Juice	4.7	3.5

NEAC, non-enzymatic antioxidant capacity; FRAP, ferric reducing-antioxidant power; ORAC, oxygen radical absorbance capacity. *^a^* Energy adjustment was performed according to the density method. *^b^* Food and beverage groups were indicated in descending order according to the mean contribution to FRAP. *^c^* The percentage contribution of each individual food and beverage item to dietary NEAC was calculated by dividing daily NEAC from each individual food and beverage item by overall dietary NEAC.

**Table 2:** Characteristics of participants according to quartiles of dietary NEAC.

	FRAP			ORAC		
	Q1 (Lowest)	Q4 (Highest)	*p*-Value *^a^*	Q1 (Lowest)	Q4 (Highest)	*p*-Value *^a^*
Age (years)	41.6 (8.7) *^b^*	44.7 (9.7)	<0.01	41.2 (8.6)	45.0 (9.9)	<0.01
Sex (men, %)	93.3	84.2	<0.01	95.5	83.2	<0.01
Workplace (site A, %)	52.9	56.7	0.37	51.1	57.3	0.05
Body Mass Index (kg/m^2^)	23.1 (3.4)	23.4 (3.3)	0.09	23.2 (3.5)	23.4 (3.3)	0.36
Occupational Physical Activity (≥20 METs-hour/day, %)	27.7	17.4	<0.01	28.6	17.1	<0.01
Leisure-Time Physical Activity (≥10 METs-hour/week, %)	24.6	26.8	<0.01	23.9	28.1	<0.01
Current Smoker (%)	34.6	25.5	<0.01	37.1	24.3	<0.01
Current Alcohol Drinker (≥23 g of ethanol/day, %)	25.2	26.6	0.38	34.2	24.3	<0.01
Dyslipidemia (%)	2.5	6.9	<0.01	2.7	5.6	0.01
Use of Anti-Inflammatory Drug (%)	7.4	8.9	0.65	6.9	8.8	0.52
Use of Antioxidant Supplements (%)	9.4	12.7	0.07	8.9	12.6	0.07
Serum Ferritin (μg/L)	138 (84, 217)	129 (70, 228)	0.045	141 (90, 218)	129 (67, 229)	0.03
Coffee Intake (≥1 cup/day, %)	73.7	59.8	<0.01	72.8	60.5	<0.01
Total Energy Intake (kcal/day)	1845 (496)	1658 (446)	<0.01	1864 (502)	1665 (451)	<0.01
Vitamin C (mg/1000 kcal) *^c^*	33.0 (14.2)	68.4 (27.4)	<0.01	29.9 (12.0)	71.2 (26.3)	<0.01
α-Tocopherol Intake (mg/1000 kcal)	3.2 (0.8)	3.9 (1.1)	<0.01	3.0 (0.8)	4.0 (1.0)	<0.01
α-Carotene Intake (μg/1000 kcal)	129 (105)	189 (164)	<0.01	112 (88.0)	204 (166)	<0.01
β-Carotene Intake (μg/1000 kcal)	1051 (662)	1730 (1174)	<0.01	928 (543)	1852 (1181)	<0.01
Cryptoxanthin (μg/1000 kcal)	75 (74)	158 (162)	<0.01	68 (69)	162 (156)	<0.01

NEAC, non-enzymatic antioxidant capacity; FRAP, ferric reducing-antioxidant power; ORAC, oxygen radical absorbance capacity; Q, quartile of dietary NEAC; Q1, <25th percentile and Q4, ≥75th percentile. *^a^* Linear regression analysis or Wilcoxon rank-sum test for continuous variables and Mantel–Haenszel test for categorical variables. *^b^* Values are mean (standard deviation) or median (25th percentile, 75th percentile) for continuous variables and percentage for categorical variables. *^c^* Ascorbic acid.

**Table 3:** Geometric means (95% CI) of serum liver enzyme levels according to quartiles (Q) of overall dietary NEAC and NEAC from foods and beverages.

	Q1 (lowest)	Q2	Q3	Q4 (highest)	*p* for Trend *^a^*
FRAP (mmol Fe^2+^) *^b^*					
Total NEAC	0.32–2.03	2.04–3.07	3.08–5.01	5.02–25.37	
Participants, n	448	446	449	448	
AST	22.1 (21.6–22.7) *^c^*	22.3 (21.7–22.8)	22.6 (22.1–23.2)	22.0 (21.4–22.6)	0.97
ALT	22.5 (21.7–23.5)	22.8 (21.9–23.7)	23.3 (22.4–24.2)	22.6 (21.7–23.5)	0.73
GGT	30.8 (29.2–32.4)	33.7 (32.0–35.5)	32.3 (30.7–34.0)	31.2 (29.6–32.9)	0.96
NEAC from Foods	0.13–0.65	0.66–0.89	0.90–1.15	1.16–3.77	
Participants, n	446	454	443	448	
AST	22.1 (21.6–22.7)	22.5 (21.9–23.0)	22.3 (21.8–22.9)	22.1 (21.6–22.7)	0.90
ALT	23.0 (22.1–23.9)	22.7 (21.9–23.6)	23.3 (22.4–24.3)	22.2 (21.3–23.1)	0.39
GGT	33.7 (32.0–35.6)	32.3 (30.7–34.0)	32.6 (31.0–34.3)	29.4 (27.9–31.0)	0.001
NEAC from Beverages	0.00–1.15	1.16–2.11	2.12–3.90	3.91–23.65	
Participants, n	450	446	446	449	
AST	22.2 (21.7–22.8)	22.3 (21.8–22.9)	22.6 (22.0–23.2)	21.9 (21.3–22.4)	0.56
ALT	22.6 (21.7–23.5)	22.7 (21.8–23.6)	23.3 (22.4–24.2)	22.7 (21.9–23.7)	0.58
GGT	30.9 (29.3–32.5)	32.8 (31.2–34.5)	33.2 (31.6–35.0)	31.1 (29.5–32.7)	0.76
ORAC (mmol TE) *^b^*					
Total NEAC	0.43–3.10	3.11–4.71	4.72–7.35	7.36–37.40	
Participants, n	448	448	450	445	
AST	22.2 (21.6–22.7)	22.2 (21.7–22.8)	22.7 (22.2–23.3)	21.9 (21.3–22.4)	0.72
ALT	22.5 (21.7–23.5)	22.9 (22.0–23.8)	23.2 (22.3–24.1)	22.5 (21.6–23.4)	0.92
GGT	31.7 (30.1–33.4)	33.6 (32.0–35.4)	32.1 (30.5–33.8)	30.5 (28.9–32.2)	0.19
NEAC from Foods	0.03–1.41	1.42–1.95	1.96–2.60	2.61–7.95	
Participants, n	456	443	452	450	
AST	22.0 (21.5–22.6)	22.5 (21.9–23.0)	22.7 (22.1–23.2)	21.8 (21.3–22.4)	0.71
ALT	22.7 (21.8–23.6)	23.2 (22.3–24.1)	23.4 (22.5–24.3)	21.9 (21.1–22.8)	0.32
GGT	32.6 (30.9–34.3)	33.0 (31.3–34.7)	33.0 (31.3–34.7)	29.6 (28.1–31.2)	0.02
NEAC from Beverages	0.00–1.18	1.19–2.46	2.47–4.99	5.00–32.33	
Participants, n	450	443	450	448	
AST	22.2 (21.7–22.8)	22.4 (21.9–23.0)	22.5 (22.0–23.1)	21.9 (21.3–22.4)	0.45
ALT	22.4 (21.6–23.3)	22.9 (22.0–23.8)	23.3 (22.4–24.3)	22.5 (21.6–23.4)	0.76
GGT	31.6 (30.0–33.2)	32.5 (30.8–34.2)	33.3 (31.7–35.1)	30.7 (29.1–32.3)	0.63

NEAC, non-enzymatic antioxidant capacity; FRAP, ferric reducing-antioxidant power; ORAC, oxygen radical absorbance capacity. Q, quartile of NEAC; Q1, <25th percentile; Q2, ≥25th percentile and <50th percentile; Q3, ≥50th percentile and <75th percentile; Q4, ≥75th percentile. *^a^* Trend tests were performed by including the ordinal numbers 1 to 3 assigned to each quartile category of dietary NEAC in a multiple linear regression analysis. *^b^* Energy adjustment was performed according to the density method. *^c^* Adjusted for age (y, continuous), sex, workplace (site A or site B), body mass index (kg/m^2^, continuous), occupational physical activity (<3, 3 to <7, 7 to <20, or ≥20 METs-hour/day), leisure-time physical activity (0, >0 to <3, 3 to <10, or ≥10 METs-hour/week), smoking status (never smoker, former smoker, current smoker of <20 cigarettes/day, or current smoker of ≥20 cigarettes/day), alcohol drinking (infrequent drinker consuming alcohol less than once per week, drinker consuming <23 g of ethanol/day, drinker consuming ≥23 to <46 g of ethanol/day, or drinker consuming ≥46 g of ethanol/day), dyslipidemia (yes or no), use of non-steroidal anti-inflammatory drugs (yes or no), log-transformed serum ferritin (μg/L, continuous), use of supplements (vitamin C, vitamin E, or multivitamins; yes or no), coffee intake (<1, 1 or ≥2 cups/day), and total energy intake (kcal/day, continuous).

**Table 4:** Geometric means (95% CI) of serum liver enzyme levels according to quartiles (Q) of dietary overall NEAC estimated by FRAP, stratified risk factors of liver injury.

	Q1 (Lowest)	Q2	Q3	Q4 (Highest)	*p* for Trend *^a^*
Age					
<Median *^b^*	248	328	172	180	
AST	21.3 (20.6–22.0) *^c^*	21.6 (20.9–22.3)	22.1 (21.3–23.0)	22.1 (21.3–23.0)	0.48
ALT	22.3 (21.1–23.5)	22.2 (21.0–23.5)	22.4 (21.0–23.8)	21.7 (20.4–23.8)	0.60
GGT	27.7 (26.0–29.5)	28.8 (27.0–30.8)	27.7 (25.7–29.9)	26.4 (24.4–28.4)	0.30
≥Median	200	208	277	268	
AST	22.8 (21.9–23.7)	23.0 (22.1–23.9)	23.2 (22.4–24.0)	22.5 (21.7–23.3)	0.70
ALT	22.6 (21.3–24.0)	23.5 (22.2–24.8)	24.0 (22.8–25.2)	23.3 (22.2–24.5)	0.40
GGT	33.3 (30.7–36.1)	38.9 (35.9–42.1)	36.8 (34.4–39.5)	36.1 (33.6–38.8)	0.36
Body Mass Index					
<25.0 kg/m^2^, n	341	345	332	327	
AST	21.1 (20.5–21.7)	21.4 (20.8–22.0)	22.1 (20.9–22.1)	21.5 (20.8–22.1)	0.21
ALT	20.2 (19.3–21.1)	20.2 (19.3–21.1)	21.1 (20.2–22.1)	20.8 (19.9–21.8)	0.21
GGT	27.5 (25.9–29.1)	29.7 (28.1–31.5)	29.6 (27.9–31.4)	28.7 (27.1–30.5)	0.33
≥25.0 kg/m^2^, n	107	101	117	121	
AST	25.3 (23.9–26.8)	25.1 (23.7–26.6)	24.5 (23.2–25.9)	23.9 (22.6–25.2)	0.14
ALT	30.8 (28.1–33.8)	32.8 (29.8–36.1)	31.5 (28.8–34.4)	29.7 (27.2–32.4)	0.46
GGT	43.3 (38.5–48.7)	49.8 (44.2–56.2)	42.0 (37.5–46.9)	40.7 (36.4–45.5)	0.19
Smoking					
Non-Smoker, n	293	307	331	334	
AST	22.0 (21.3–22.6)	22.3 (21.6–23.0)	22.9 (22.2–23.6)	22.0 (21.4–22.7)	0.59
ALT	22.0 (20.9–23.1)	22.3 (21.2–23.3)	22.9 (21.9–23.9)	21.9 (21.0–23.0)	0.85
GGT	29.9 (28.0–31.9)	32.1 (30.2–34.2)	31.0 (29.2–32.9)	29.1 (27.3–30.9)	0.40
Smoker, n	155	139	118	114	
AST	22.5 (21.6–23.4)	22.0 (21.1–23.0)	22.1 (21.1–23.2)	21.8 (20.8–22.9)	0.42
ALT	24.0 (22.4–25.8)	23.8 (22.2–25.6)	24.5 (22.6–26.5)	24.3 (22.4–26.3)	0.76
GGT	33.8 (31.0–36.8)	37.3 (34.1–40.7)	36.2 (32.9–39.9)	37.0 (33.5–40.9)	0.22
Alcohol Drinking					
<23 g of ethanol/day, n	335	324	307	329	
AST	21.4 (20.8–22.0)	21.5 (20.9–22.2)	22.2 (21.5–22.8)	21.4 (20.8–22.0)	0.68
ALT	22.4 (21.4–23.5)	22.2 (21.2–23.3)	23.0 (21.9–24.1)	22.3 (21.3–23.4)	0.92
GGT	27.5 (26.0–29.1)	29.1 (27.4–30.8)	28.1 (26.4–29.8)	26.5 (25.0–28.1)	0.30
≥23 g of ethanol/day, n	113	122	142	119	
AST	24.0 (22.8–25.4)	24.4 (23.1–25.6)	24.1 (23.0–25.3)	23.8 (22.5–25.1)	0.73
ALT	22.6 (20.9–24.5)	24.1 (22.4–26.0)	24.1 (22.5–25.9)	23.7 (21.9–25.6)	0.42
GGT	40.7 (36.2–45.8)	50.4 (45.0–56.4)	46.7 (42.1–51.8)	47.3 (42.1–53.2)	0.18
Ferritin					
<Median *^d^*, n	248	220	208	224	
AST	21.2 (20.5–21.8)	20.6 (19.9–21.3)	21.6 (20.9–22.3)	20.6 (20.0–21.3)	0.65
ALT	21.2 (20.2–22.2)	19.8 (18.8–20.9)	21.3 (20.2–22.4)	20.1 (19.1–21.2)	0.43
GGT	27.4 (25.7–29.3)	28.0 (26.1–29.9)	28.8 (26.8–30.9)	26.9 (25.1–28.8)	0.87
≥Median, n	200	226	241	224	
AST	23.0 (22.0–23.9)	24.1 (23.2–25.0)	23.8 (22.9–24.7)	23.5 (22.6–24.5)	0.57
ALT	23.7 (22.3–25.3)	25.5 (24.0–27.0)	25.6 (24.1–27.3)	25.6 (24.1–27.3)	0.18
GGT	34.3 (31.6–37.3)	40.5 (37.5–43.8)	36.2 (33.6–39.1)	36.7 (33.9–39.8)	0.69

NEAC, non-enzymatic antioxidant capacity; FRAP, ferric reducing-antioxidant power. Q, quartile of NEAC; Q1, <25th percentile; Q2, ≥25th percentile and <50th percentile; Q3, ≥50th percentile and <75th percentile; Q4, ≥75th percentile. *^a^* Trend tests were performed by including the ordinal numbers 1 to 3 assigned to each quartile category of dietary NEAC in a multiple linear regression analysis. *^b^* The median age was 42 years old. *^c^* Adjusted for age (y, continuous), sex, workplace (site A or B), body mass index (kg/m^2^, continuous), occupational physical activity (<3, 3 to <7, 7 to <20, or ≥20 METs-hour/day), leisure-time physical activity (0, >0 to <3, 3 to <10, or ≥10 METs-hour/week), smoking status (never smoker, former smoker, current smoker of <20 cigarettes/day, or current smoker of ≥20 cigarettes/day), alcohol drinking (infrequent drinker consuming alcohol less than once per week, drinker consuming <23 g of ethanol/day, drinker consuming ≥23 to <46 g of ethanol/day, or drinker consuming ≥46 g of ethanol/day), dyslipidemia (yes or no), use of non-steroidal anti-inflammatory drugs (yes or no), log-transformed serum ferritin (μg/L, continuous), use of supplements (vitamin C, vitamin E, or multivitamins; yes or no), coffee intake (<1, 1 or ≥2 cups/day), and total energy intake (kcal/day, continuous), except for the stratified variable. *^d^* Median serum ferritin level was 155 μg/L for men and 23 μg/L for women. There was no evidence of an interaction between overall dietary NEAC estimated by FRAP and any confounding factors on serum liver enzymes (*p* >0.05).

## Supplementary Materials

The following are available online at https://www.mdpi.com/2072-6643/12/7/2051/s1, Figure S1: The main contribution of food items according to quartiles (Q) of dietary non-enzymatic antioxidant capacity estimated by ferric reducing-antioxidant power (FRAP) and oxygen radical absorbance capacity (ORAC), Table S1: Geometric means (95% CI) of serum liver enzyme levels according to quartiles (Q) of dietary overall NEAC estimated by ORAC, stratified risk factors of liver injury, Table S2: Geometric means (95% CI) of serum liver enzyme levels according to quartiles (Q) of dietary NEAC from foods estimated by FRAP, stratified risk factors of liver injury, Table S3: Geometric means (95% CI) of serum liver enzyme levels according to quartiles (Q) of dietary NEAC from foods estimated by ORAC, stratified risk factors of liver injury.
