# Supplementary material for: Cross-Sectional Study on the Association between Dietary Non-Enzymatic Antioxidant Capacity and Serum Liver Enzymes: The Furukawa Nutrition and Health Study
Source: Nutrients. 2020 Jul 10;12(7):2051. doi: 10.3390/nu12072051 (PMC7400899; doi:10.3390/nu12072051)
Supplement: Supplementary file 1 [file nutrients-12-02051-s001.pdf]

Supplemental Figure Legend.

Supplemental Figure S1. The main contribution of food items according to quartiles (Q) of dietary non-enzymatic antioxidant capacity estimated by ferric reducing-antioxidant power (FRAP) and oxygen radical absorbance capacity (ORAC).

Based on the overall NEAC quartiles, beverages, particularly green tea, made the greatest contribution to the total NEAC intake in each quartile, with the exception of the first quartile.

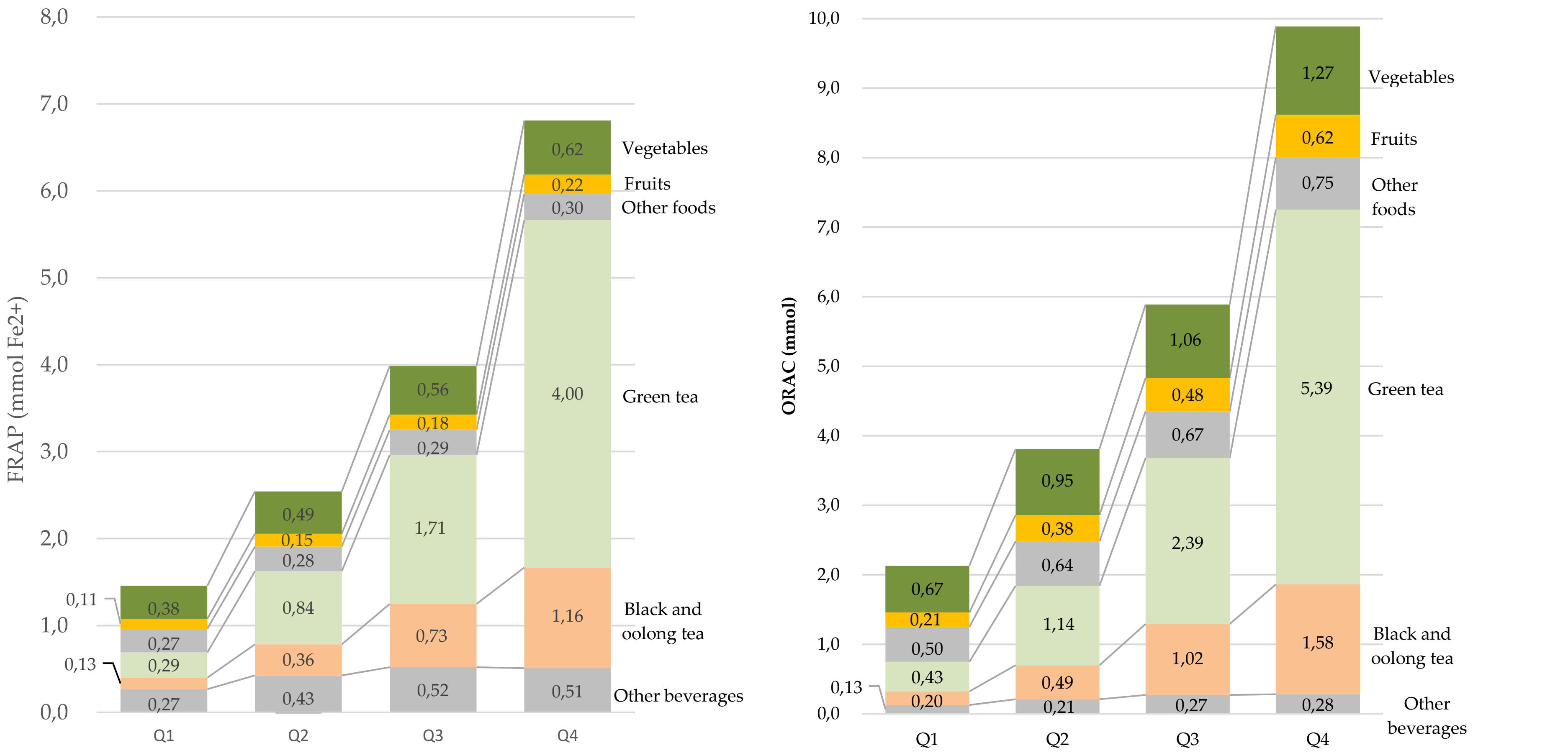

Supplemental Figure S1. The main contribution of food items according to quartiles (Q) of dietary non-enzymatic antioxidant capacity estimated by ferric reducing-antioxidant power (FRAP) and oxygen radical absorbance capacity (ORAC). Other foods; the sum of cereals, potatoes, pulses, confectionary and oil, other beverages; the sum of fruit juice and vegetable juice, and alcoholic beverages.

Supplemental Table S1. Geometric means (95% CI) of serum liver enzyme levels according to quartiles (Q) of dietary overall NEAC estimated by ORAC, stratified risk factors of liver injury.

	Q1 (lowest)	Q2	Q3	Q4 (highest)	*p* for Trend [a]
Age					
<Median [b]	251	229	181	177	
AST	21.5 (20.9–22.2) [c]	21.3 (20.6–22.1)	22.1 (21.3–22.9)	21.4 (20.6–22.3)	0.77
ALT	22.5 (21.3–23.7)	22.1 (20.9–23.4)	22.4 (21.0–23.8)	21.5 (20.1–22.9)	0.37
GGT	24.5 (26.7–30.3)	28.5 (26.7–30.4)	27.3 (25.4–29.4)	26.1 (24.2–28.2)	0.08
≥Median	197	219	269	268	
AST	22.7 (21.8–23.6)	23.0 (22.2–23.9)	23.3 (22.6–24.1)	22.4 (21.6–23.1)	0.62
ALT	22.5 (21.2–23.8)	23.7 (22.5–25.1)	23.9 (22.7–25.1)	23.3 (22.1–24.5)	0.43
GGT	34.5 (31.8–37.5)	38.8 (35.9–41.9)	37.0 (34.5–39.7)	34.9 (32.5–37.5)	0.80
Body mass index					
<25.0 kg/m^2, n	336	352	328	329	
AST	21.2 (20.6–21.8)	21.4 (20.8–22.0)	22.1 (21.5–22.7)	22.3 (20.7–21.9)	0.51
ALT	20.2 (19.3–21.1)	20.5 (19.6–21.4)	20.8 (19.8–21.7)	20.8 (19.9–21.8)	0.32
GGT	28.5 (26.9–30.3)	29.6 (28.0–31.4)	29.2 (27.5–30.9)	28.1 (26.5–29.9)	0.68
≥25.0 kg/m^2, n	112	96	122	116	
AST	25.1 (23.7–26.6)	25.3 (23.9–26.9)	24.6 (23.3–26.0)	23.8 (22.5–25.2)	0.16
ALT	30.8 (28.0–33.8)	32.2 (30.1–36.6)	31.6 (29.0–34.5)	29.3 (26.8–32.0)	0.36
GGT	42.9 (38.2–48.2)	52.2 (46.2–58.9)	42.0 (37.7–46.8)	39.6 (35.4–44.3)	0.10
Smoking					
Non-smoker, n	282	307	339	337	
AST	22.2 (21.5–22.9)	22.1 (21.5–22.8)	22.9 (22.3–23.6)	22.0 (21.3–22.6)	0.97
ALT	22.2 (21.1–23.3)	22. 1(21.1–23.2)	22.8 (21.8–23.8)	21.9 (20.9-22.9)	0.92
GGT	31.5 (29.5–33.7)	31.4 (29.5–33.7)	31.0 (29.2–33.0)	28.3 (26.6–30.1)	0.07
Smoker, n	166	141	111	108	
AST	22.2 (21.4–23.2)	22.2 (21.3–23.2)	22.4 (21.3–23.5)	21.7 (20.6–22.8)	0.56
ALT	23.6 (22.0–25.8)	24.6 (22.9–26.4)	24.5 (22.6–26.6)	24.1 (22.1–26.2)	0.68
GGT	33.5 (30.8–36.4)	38.9 (35.6–42.5)	35.2 (31.8–38.8)	36.8 (33.2–40.7)	0.34
Alcohol drinking					
<23 g of ethanol/day, n	295	327	336	337	
AST	21.6 (20.9–22.2)	21.5 (20.9–22.2)	22.0 (21.4–22.7)	21.2 (20.6–21.9)	0.72
ALT	22.3 (21.2–23.4)	22.5 (21.5–23.5)	22.8 (21.8–23.9)	22.3 (21.2–23.3)	0.94
GGT	27.8 (26.1–29.5)	29.5 (27.9–31.3)	27.8 (26.2–29.4)	26.2 (24.7–27.7)	0.07
≥23 g of ethanol /day, n	153	121	114	108	
AST	24.1 (23.0–25.2)	24.2 (23.0–25.5)	24.5 (23.2–25.8)	23.5 (22.2–24.8)	0.65
ALT	23.3 (21.8–24.9)	23.9 (22.2–25.7)	24.2 (22.4–26.2)	23.4 (21.6–25.4)	0.80
GGT	46.0 (41.6–51.0)	47.8 (42.7–53.5)	46.5 (41.4–52.3)	44.6 (39.4–50.5)	0.71
Ferritin					
<Median [d], n	248	224	209	219	
AST	21.2 (20.5–21.8) [d]	20.6 (20.0–21.3)	21.6 (20.9–22.3)	20.6 (20.0–21.3)	0.98
ALT	21.1 (20.1–22.2)	21.0 (19.1–21.1)	21.0 (19.9–22.1)	20.2 (19.1–21.3)	0.33<br>33
GGT	28.4 (26.6–30.3)	27.7 (25.9–29.6)	28.1 (26.2–30.2)	26.7 (24.9–28.6)	0.08
≥Median, n	200	224	241	226	
AST	23.2 (22.3–24.2)	24.0 (23.1–24.9)	24.9 (23.0–24.9)	23.9 (23.0–24.8)	0.67
ALT	23.9 (22.4–25.5)	26.2 (24.6–27.8)	25.5 (24.1–27.1)	25.3 (23.8–27.0)	0.30
GGT	35.3 (32.5–38.4)	40.8 (37.7–44.2)	36.5 (33.8–39.4)	35.2 (32.5–38.2)	0.98

NEAC, non-enzymatic antioxidant capacity; FRAP, ferric reducing-antioxidant power. Q, quartile of NEAC; Q1, <25th percentile; Q2, ≥25th percentile and <50th percentile; Q3, ≥50th percentile and <75th percentile; Q4, ≥75th percentile. [a] Trend tests were performed by including the ordinal numbers 1 to 3 assigned to the each quartile category of dietary NEAC in a multiple linear regression analysis. [b] The median age was 42 years old. [c] Adjusted for age (y, continuous), sex, workplace (site A or B), body mass index (kg/m^2, continuous), occupational physical activity (<3, 3 to <7, 7 to <20, or ≥20 METs-hour/day), leisure-time physical activity (0, >0 to <3, 3 to <10, or ≥10 METs-hour/week), smoking status (never smoker, former smoker, current smoker of <20 cigarettes/day, or current smoker of ≥20 cigarettes/day), alcohol drinking (infrequent drinker consuming alcohol

less than once per week, drinker consuming <23 g of ethanol/day, drinker consuming ≥23 to <46 g of ethanol/day, or drinker consuming ≥46 g of ethanol/day), dyslipidemia (yes or no), use of non-steroidal anti-inflammatory drugs (yes or no), log-transformed serum ferritin (μg/L, continuous), use of supplements (vitamin C, vitamin E, or multivitamins; yes or no), coffee intake (<1, 1 or ≥2 cups/day), and total energy intake (kcal/day, continuous), except for the stratified variable. [d] Median serum ferritin level was 155 μg/L for men and 23 μg/L for women. There were no evidence of an interaction between overall dietary NEAC estimated by ORAC and any confounding factors on serum liver enzymes ($p > 0.05$).

Supplemental Table S2. Geometric means (95% CI) of serum liver enzyme levels according to quartiles (Q) of dietary NEAC from foods estimated by FRAP, stratified risk factors of liver injury.

	Q1 (lowest)	Q2	Q3	Q4 (highest)	*p* for Trend [a]
Age					
<Median [b], n	240	239	194	165	
AST	21.2 (20.5–21.9) [c]	21.6 (20.9–22.3)	21.5 (20.7–22.3)	22.3 (21.4–23.2)	0.12
ALT	21.9 (20.7–23.1)	22.2 (21.0–23.5)	22.3 (21.0–23.7)	22.4 (20.9–23.9)	0.62
GGT	28.4 (26.5–30.3)	28.2 (26.4–30.1)	26.6 (24.8–28.7)	27.4 (25.3–29.6)	0.32
≥Median, n	206	215	249	283	
AST	22.9 (22.0–23.8)	23.3 (22.4–24.2)	23.0 (22.2–23.8)	22.4 (21.7–23.2)	0.33
ALT	24.2 (22.8–25.6)	23.3 (22.0–24.6)	24.1 (22.9–25.4)	22.3 (21.2–23.4)	0.08
GGT	39.5 (36.3–42.8)	36.4 (33.6–39.3)	38.7 (36.0–41.7)	32.2 (30.0–34.5)	0.001*
Body mass index					
<25.0 kg/m², n	320	347	339	339	
AST	21.4 (20.8–22.1)	21.6 (21.1–22.2)	21.5 (20.9–22.1)	21.4 (20.8–22.0)	0.85
ALT	20.8 (19.8–21.8)	20.5 (19.6–21.4)	21.1 (20.2–22.0)	20.0 (19.1–20.9)	0.42
GGT	30.8 (29.0–32.8)	29.2 (27.6–30.9)	29.2 (27.6–31.0)	26.5 (25.0–28.1)	0.002
≥25.0 kg/m², n	126	107	104	109	
AST	24.4 (23.1–25.8)	25.5 (24.1–27.1)	24.7 (23.3–26.2)	24.1 (22.7–25.5)	0.60
ALT	31.2 (28.5–34.1)	32.5 (29.7–35.7)	31.7 (28.8–34.8)	29.2 (26.6–32.0)	0.29
GGT	44.8 (40.0–50.1)	45.7 (40.6–51.4)	45.4 (40.3–51.1)	38.8 (34.5–43.7)	0.11
Smoking					
Non-smoker, n	257	317	329	362	
AST	22.2 (21.5–23.0)	22.5 (21.8–23.2)	22.2 (21.6–22.9)	22.2 (21.6–22.9)	0.86
ALT	22.4 (21.3–23.6)	22.6 (21.6–23.7)	22.5 (21.5–23.5)	21.6 (20.7–22.6)	0.26
GGT	32.4 (30.2–34.8)	31.7 (29.8–33.7)	30.6 (28.8–32.6)	28.1 (26.5–29.8)	0.001
Smoker, n	189	137	114	86	
AST	22.0 (21.2–22.9)	22.2 (21.2–23.2)	22.6 (21.5–23.7)	21.8 (20.6–23.0)	0.99
ALT	24.4 (22.9–26.0)	22.9 (21.3–24.6)	25.8 (23.8–28.0)	23.5 (21.4–25.8)	0.91
GGT	37.8 (34.9–40.8)	34.0 (31.1–37.2)	38.1 (34.5–42.1)	32.4 (28.9–36.4)	0.15
Alcohol drinking					
<23 g of ethanol/day, n	277	319	344	355	
AST	21.5 (20.8–22.2)	21.8 (21.2–22.4)	21.7 (21.1–22.3)	21.4 (20.8–22.0)	0.85
ALT	22.7 (21.6–23.9)	22.6 (21.6–23.7)	22.8 (21.8–23.9)	21.8 (20.9–22.8)	0.30
GGT	30.3 (28.4–32.3)	28.8 (27.1–30.5)	27.6 (26.1–29.2)	25.3 (23.9–26.7)	<0.001
≥23 g of ethanol /day, n	169	135	99	93	
AST	24.2 (23.1–25.3)	24.3 (23.1–25.5)	23.8 (22.5–25.5)	23.9 (22.5–25.3)	0.61
ALT	23.8 (22.3–25.5)	23.3 (21.7–25.0)	24.5 (22.5–26.6)	23.1 (21.2–25.3)	0.86
GGT	47.1 (42.7–52.0)	44.7 (40.2–49.7)	49.3 (43.5–55.9)	43.9 (38.5–50.1)	0.74
Ferritin					
<Median [d], n	250	214	225	211	
AST	20.8 (20.1–21.4) [d]	21.0 (20.3–21.7)	20.9 (20.3–21.7)	21.4 (20.7–22.1)	0.26
ALT	23.6 (22.6–24.6)	24.0 (23.1–24.9)	23.7 (22.7–24.6)	23.1 (22.2–24.0)	0.96
GGT	20.7 (19.7–21.8)	20.2 (19.2–21.3)	20.8 (19.8–21.9)	20.6 (19.5–21.7)	0.16
≥Median, n	196	240	218	237	
AST	25.3 (23.7–27.1)	25.7 (24.2–27.2)	26.0 (24.4–27.6)	24.2 (22.8–25.7)	0.35
ALT	29.0 (27.1–30.9)	27.6 (25.7–29.5)	27.2 (25.5–29.1)	27.0 (25.2–29.0)	0.36
GGT	39.6 (36.3–43.2)	37.7 (35.0–40.7)	38.6 (35.7–41.8)	32.7 (30.3–35.4)	0.004*

NEAC, non-enzymatic antioxidant capacity; FRAP, ferric reducing-antioxidant power. Q, quartile of NEAC; Q1, <25th percentile; Q2, ≥25th percentile and <50th percentile; Q3, ≥50th percentile and <75th percentile; Q4, ≥75th percentile. [a] Trend tests were performed by including the ordinal numbers 1 to 3 assigned to the each quartile category of dietary NEAC in a multiple linear regression analysis. [b] The median age was 42 years old. [c] Adjusted for age (y, continuous), sex, workplace (site A or B), body mass index (kg/m², continuous), occupational physical activity (<3, 3 to <7, 7 to <20, or ≥20 METs-hour/day), leisure-time physical activity (0, >0 to <3, 3 to <10, or ≥10 METs-hour/week), smoking status (never smoker, former smoker, current smoker of <20 cigarettes/day, or current smoker of ≥20 cigarettes/day), alcohol drinking (infrequent drinker consuming alcohol less than once per week, drinker consuming <23 g of ethanol/day, drinker consuming ≥23 to <46 g of ethanol/day, or drinker consuming ≥46 g of ethanol/day), dyslipidemia (yes or no), use of non-steroidal anti-inflammatory drugs (yes or no), log-transformed serum ferritin (μg/L, continuous), use of supplements (vitamin C, vitamin E, or multivitamins; yes or no), coffee intake (<1, 1 or ≥2 cups/day), and total energy intake (kcal/day, continuous), except for the stratified variable. [d] Median serum ferritin level is 155 μg/L for men and 23 μg/L for

women. * Significantly interaction between dietary NEAC from foods estimated by FRAP and age group (<median or ≥median) or serum ferritin level (<median or ≥median) on serum GGT: * $p < 0.05$. There were no evidence of an interaction between dietary NEAC from foods estimated by FRAP and other confounding factors on serum liver enzymes ($p > 0.05$).

Supplemental Table S3. Geometric means (95% CI) of serum liver enzyme levels according to quartiles (Q) of dietary NEAC from foods estimated by ORAC, stratified risk factors of liver injury.

	Q1 (lowest)	Q2	Q3	Q4 (highest)	*p* for Trend [a]
Age					
<Median [b]	243	232	196	167	
AST	21.3 (20.6–22.0) [c]	21.5 (20.8–22.3)	21.6 (20.8–22.4)	22.0 (21.2–22.9)	0.22
ALT	21.8 (20.6–23.1)	22.7 (21.4–24.0)	21.8 (20.5–23.2)	22.3 (20.9–23.9)	0.85
GGT	28.1 (26.3–30.1)	28.3 (26.5–30.3)	26.5 (24.7–28.5)	27.7 (25.6–30.0)	0.46
≥Median	203	211	256	283	
AST	22.7 (21.8–23.6)	23.3 (22.5–24.2)	23.5 (22.7–24.3)	22.1 (21.4–22.9)	0.28
ALT	23.7 (22.3–25.1)	23.8 (22.5–25.2)	24.5 (23.3–25.8)	21.9 (20.9–23.1)	0.07
GGT	36.9 (34.0–40.1)	37.9 (35.0–41.1)	39.2 (36.4–42.1)	32.3 (30.1–34.6)	0.02
Body mass index					
<25.0 kg/m^2, n	318	332	353	342	
AST	21.5 (20.9–22.2)	21.6 (21.0–22.2)	21.8 (21.2–22.4)	21.1 (20.5–21.7)	0.46
ALT	20.7 (29.0–32.8)	29.2 (27.6–30.9)	29.2 (27.6–31.0)	26.5 (25.0–28.1)	0.22
GGT	30.4 (28.5–32.3)	29.3 (27.7–31.1)	29.6 (28.0–31.3)	26.4 (24.9–28.0)	0.004
≥25.0 kg/m^2, n	128	111	99	108	
AST	24.0 (22.7–25.3)	25.5 (24.1–27.0)	25.5 (24.1–27.0)	25.5 (24.0–27.0)	0.95
ALT	30.8 (28.2–33.6)	32.4 (29.6–35.4)	32.2 (29.2–35.4)	29.4 (26.7–32.3)	0.52
GGT	41.6 (37.2–46.5)	47.4 (42.2–53.1)	45.7 (40.5–51.7)	40.5 (35.9–45.6)	0.72
Smoking					
Non-smoker, n	256	304	348	357	
AST	22.1 (21.4–22.9)	22.5 (21.8–23.2)	22.7 (22.1–23.3)	21.9 (21.3–22.5)	0.61
ALT	22.2 (21.0–23.3)	22.8 (21.8–23.9)	22.8 (21.8–23.8)	21.3 (20.4–22.3)	0.24
GGT	31.4 (29.2–33.6)	31.8 (29.9–33.9)	31.3 (29.5–33.2)	28.1 (26.5–29.8)	0.01
Smoker, n	190	139	104	93	
AST	21.9 (21.1–22.8)	22.3 (21.4–23.3)	22.5 (21.4–23.7)	21.9 (20.8–23.1)	0.80
ALT	24.0 (22.5–25.6)	24.0 (22.3–25.8)	25.0 (22.9–27.2)	23.7 (21.7–26.0)	0.92
GGT	36.3 (33.6–39.3)	35.8 (32.8–39.1)	37.7 (34.0–41.8)	33.3 (29.8–37.3)	0.44
Alcohol drinking					
<23 g of ethanol/day, n	280	316	339	360	
AST	21.6 (20.9–22.3)	21.5 (20.9–22.2)	22.1 (21.5–22.7)	21.2 (20.6–21.8)	0.56
ALT	22.6 (21.5–23.8)	22.7 (21.6–23.8)	23.2 (22.2–24.3)	21.6 (20.6–22.6)	0.25
GGT	28.9 (27.2–30.8)	29.2 (27.6–31.0)	27.9 (26.4–29.5)	25.6 (24.2–27.1)	0.002
≥23 g of ethanol /day, n	166	127	133	90	
AST	23.8 (22.7–24.9)	24.8 (23.6–26.0)	24.1 (22.9–25.5)	23.5 (22.1–25.0)	0.72
ALT	23.6 (22.0–25.2)	24.3 (22.6–26.2)	23.7 (22.0–25.7)	22.9 (21.0–25.0)	0.62
GGT	46.3 (41.8–51.2)	45.1 (40.4–50.3)	49.8 (44.3–56.0)	43.6 (38.2–49.9)	0.89
Ferritin					
<Median [d], n	253	206	233	208	
AST	20.6 (20.0–21.3) [d]	21.2 (20.5–21.9)	21.1 (20.5–21.8)	21.1 (20.4–21.8)	0.35
ALT	20.3 (19.3–21.3)	20.7 (19.7–21.8)	21.1 (20.1–22.2)	20.3 (19.2–21.4)	0.84
GGT	28.5 (26.7–30.5)	28.2 (26.3–30.2)	27.5 (25.8–29.4)	26.6 (24.8–28.6)	0.16
≥Median, n	193	237	219	242	
AST	23.8 (22.8–24.8)	23.7 (22.8–24.6)	24.3 (23.4–25.3)	22.7 (21.9–23.6)	0.21
ALT	25.9 (24.4–27.4)	26.0 (24.4–27.6)	23.9 (22.5–27.6)	23.9 (22.5–25.4)	0.17
GGT	37.6 (34.4–41.0)	38.1 (35.3–41.2)	39.1 (36.1–42.4)	33.5 (31.0–36.3)	0.07

NEAC, non-enzymatic antioxidant capacity; ORAC, oxygen radical absorbance capacity. Q, quartile of NEAC; Q1, <25th percentile; Q2, ≥25th percentile and <50th percentile; Q3, ≥50th percentile and <75th percentile; Q4, ≥75th percentile. [a] Trend tests were performed by including the ordinal numbers 1 to 3 assigned to the each quartile category of dietary NEAC in a multiple linear regression analysis. [b] The median age is 42 years old. [c] Adjusted for age (y, continuous), sex, workplace (site A or B), body mass index (kg/m^2, continuous), occupational physical activity (<3, 3 to <7, 7 to <20, or ≥20 METs-hour/day), leisure-time physical activity (0, >0 to <3, 3 to <10, or ≥10 METs-hour/week), smoking status (never smoker, former smoker, current smoker of <20 cigarettes/day, or current smoker of ≥20 cigarettes/day), alcohol drinking (infrequent drinker consuming alcohol less than once per week, drinker consuming <23 g of ethanol/day, drinker consuming ≥23 to <46 g of ethanol/day, or drinker consuming ≥46 g of ethanol/day), dyslipidemia (yes or no), use of non-steroidal anti-inflammatory drugs (yes or no), log-transformed serum ferritin (μg/L, continuous), use of supplements (vitamin C, vitamin E, or multivitamins; yes or no), coffee intake (<1, 1 or ≥2 cups/day), and total energy intake (kcal/day, continuous), except for the stratified variable. [d] Median serum ferritin level is 155 μg/L for men and 23 μg/L for

women. There were no evidence of an interaction between dietary NEAC from foods estimated by ORAC and any confounding factors on serum liver enzymes (*p* >0.05).
